# 肺癌干细胞作为靶点的肺癌治疗策略研究进展

**DOI:** 10.3779/j.issn.1009-3419.2018.01.08

**Published:** 2018-01-20

**Authors:** 红锦 赖, 锋 林, 楠 陈, 舒 文, 晓 胡, 伦旭 刘

**Affiliations:** 1 610041 成都，四川大学华西医院 West China School Medicine, West China Hospital, Sichuan University, Chengdu 610041, China; 2 610041 成都，四川大学华西医院，胸外科 Department of Thoracic Surgery, West China Hospital, Sichuan University, Chengdu 610041, China; 3 550004 贵阳，贵州医科大学附属医院胸外科 Department of Thoracic Surgery, the Affiliated Hospital of Guizhou Medical University, Guiyang 550004, China

**Keywords:** 肺癌, 肺癌干细胞, 分子靶向治疗, 肿瘤干细胞, Lung neoplasms, Lung cancer stem cells, Molecular targeted therapy, Neoplastic stem cells

## Abstract

肺癌发病率高、死亡率高，严重威胁人类健康，是肿瘤研究的热点之一。肺癌干细胞是指存在于肺癌组织中的一类细胞亚群，具有自我更新及分化能力并可能导致了肿瘤发生及肿瘤异质性的产生。肺癌干细胞理论可以解释肺癌复发、转移、抗化疗药物等特性，针对这类细胞的靶向治疗有一定的运用前景。本文简要介绍肺癌干细胞的标志物、其异常的信号通路等，并概述靶向肺癌干细胞的治疗策略。

## Abstract (en)

With high morbidity and mortality, lung cancer is a major threat to human health and one of the focuses of tumor researches. Lung cancer stem cells (LCSCs) are regarded as a subpopulation of cells within lung cancer tissues with the capacity of self-renewal and differentiation, and might be related to tumorigenesis and heterogeneity of lung cancer. Tumor recurrence, metastasis and drug resistance of lung cancers could be clarified by LCSC hypothesis. Thus it's therapeutically prospective to target at these cells. This review summarizes the biomarkers of LCSCs and their aberrant signal pathways, as well as the therapeutic strategies targeting at LCSCs.

根据2016年公布的数据，在各种类型的癌症中，肺癌的发病率和死亡率均居于前列^[1]^。近30年来，肺癌5年生存率虽逐步上升，但仍然低于30%，其严峻形势不容乐观。癌症干细胞（cancer stem cell, CSC）理论认为恶性肿瘤发生可能是具有自我更新及分化能力的干细胞导致^[2, 3]^。在肺癌的发生发展过程中，肺癌干细胞可能扮演了重要的角色，在肿瘤的复发、转移、抗化疗药物等方面发挥作用^[4-8]^。本文就以肺癌干细胞（lung cancer stem cell, LCSC）为靶点的治疗策略的研究进展进行综述。

## 1 肺癌干细胞标志物

目前对于肺癌干细胞的标志物的认定尚无明确标准，但是存在一些在肺癌干细胞中广泛表达的细胞表面蛋白或酶等被广泛认可为阳性标志物，如CD133、CD166以及ALDH等^[7-9]^；此外，CD24、CD45等标志物在肺癌干细胞中呈阴性或低表达^[10]^，被认为是阴性标志物，无法作为靶点。

### 1.1 CD133

CD133也称人Prominin-1，是一种5次跨膜的糖蛋白，由865个氨基酸残基组成，分子量约97 kDa^[11]^。CD133在肝癌、脑胶质瘤、结肠癌中都被证实为癌症干细胞标志物^[8]^，且其阳性表达与LCSC关系密切。Tirino等^[12]^对非小细胞肺癌（non-small cell lung cancer, NSCLC）样本的细胞进行体外培养分析，并将培养结果与临床标本比对，研究发现CD133阳性细胞比例显著增加，提示其增殖能力强于其他细胞。Hilbe等研究^[13]^显示，NSCLC临床样本中CD133阳性细胞的增加与肿瘤组织血管生成具有相关性。Eramo等研究^[14]^结果显示，CD133阳性细胞注入SCID小鼠皮下增殖分化产生与原肿瘤表型相同的肿瘤，且CD133与Ep-CAM的共表达可能与肿瘤的血管内皮发生有关。

### 1.2 ALDH

ALDH（aldehyde dehydrogenases），即乙醛脱氢酶，负责催化乙醛等物质的氧化脱氢等重要反应，ALDH对视黄醛氧化具有极高效的催化作用，这对组织细胞的分化调控具有重要作用^[15]^。ALDH在白血病、肝癌、胰腺癌、乳腺癌、结肠癌等的癌症干细胞中都异常活跃^[8]^，可作为不良预后的指标^[16]^。Moreb等通过对不同肺癌细胞系进行流式细胞术分析，发现ALDH1A1和ALDH3A1两种亚型在某些NSCLC细胞系中高表达^[17, 18]^。Ucar等^[19]^通过对H522肺癌细胞系ALDH表达程度不同的细胞分类研究，发现高表达ALDH的一类细胞呈现出类似干细胞的特征：增殖较缓慢，可产生具形态学差异的不同集落，在NOD/SCID（非肥胖糖尿病/重症联合免疫缺陷）小鼠中的初代移植瘤生长缓慢等。而直接从NSCLC标本中分离的ALDH1阳性细胞，在体外实验与体内实验中均呈现出一系列癌症干细胞特征^[20]^。

### 1.3 ABCG2

ABCG2是隶属于ABC蛋白家族的膜转运蛋白，在正常组织广泛分布，干细胞中高表达，与肿瘤细胞多重耐药具有相关性^[21, 22]^。ABCG2在结肠癌、肝癌、肺癌等肿瘤中均可作为CSC标志物^[21-23]^。Ho等^[24]^从H460、H23、HTB-58、A549、H441、H2170六种肺癌细胞系中分离出的侧群细胞（side population, SP）均高表达ABCG2，因而ABCG2被认为是SP的表型；同时该研究证实SP在NOD/SCID小鼠中具有比非SP更强的致瘤作用，SP中端粒酶逆转录酶高表达，提示肺癌的侧群细胞具有干细胞的特性。Sung等^[25]^将从A549肺癌细胞系中分离的SP用ABCG2的选择性抑制剂烟曲霉毒素处理后，SP全部消失；利用基因芯片技术对SP的ABCG2的mRNA表达进行测定，也显示出高表达的结果。

### 1.4 CD44

CD44是一种跨膜糖蛋白，作为信号受体发挥介导细胞粘附、迁徙等多种功能，有研究表明其在结直肠癌中可作为CSC标志物^[8]^。Liu等^[26]^研究发现，CD44阳性肺癌细胞中Nanog基因高表达，而Nanog基因则在干细胞获得多潜能的过程中发挥极为重要的作用^[27]^。作为一种重要的信号受体，CD44可介导肿瘤的增殖，如作为表皮生长因子受体或ErbB家族其他受体酪氨酸激酶的共受体，间接激活细胞增殖^[8]^。Leung等^[28]^研究证实了CD44阳性NSCLC细胞在裸鼠体内分化的能力，且新分离的CD44阳性细胞比阴性细胞对顺铂治疗有着更强的抗性。

### 1.5 其他

除上述几种认可度较高的标志物外，一些研究证实，CD166也在LCSC中表达较为活跃。CD166属于细胞粘附分子免疫球蛋白超家族（Ig-CAM），介导细胞间的粘附等功能，与白细胞渗出有密切关系；Zhang等^[29]^从肺部肿瘤中分选出肿瘤起始细胞（tumor-initiating cell, TIC）亚群，小鼠体内实验结果显示CD166阳性的细胞具有比CD166阴性细胞更强的致瘤能力。

需要特别指出的是，LCSC往往存在多种标志物共表达的情况，有学者认为单个的标志物不足以作为LCSC的证据，需要多重标志物对LCSC进行定义^[8]^。

## 2 肺癌干细胞的调控信号通路

尽管积累了较多肿瘤细胞调控信号通路相关基因的变异的资料，但目前肺癌的肿瘤异质性以及耐药性的具体产生机制仍不十分清楚^[9]^。随着对以下几种信号通路与肿瘤发生、肿瘤耐药之间相关性的研究逐步深入，有理由相信，与胚胎发育或分裂分化相关的信号通路的异常激活和肿瘤的发生及恶性转移有较强联系^[8]^。

### 2.1 Notch通路

人类共有四种Notch受体，Notch信号通路在胚胎发育调控及其他多种生物功能中发挥重要作用，其变异对于CSC的产生和维持可能有着重要的促进作用。Westhoff等^[30]^发现，在NSCLC中，约1/3的样本存在着Notch通路的变异，并确定两种变异类型：Numb表达的缺失和Notch-1功能获得性突变。上皮-间充质转化（epithelial-mesenchymal transition, EMT）可使癌细胞获得干细胞样特性和耐药性等，Notch通路与多种转录因子间的交流可加强上皮-间充质转化的过程，促使NSCLC获得更高的运动性、侵袭力及代谢活力^[31]^。Hassan等^[32]^研究发现，Notch活性高的肺腺癌细胞能在无血清培养基中形成更多的肿瘤球并可分化出Notch低活性的细胞群体，其中高活性的细胞对化疗有抗性且在NOD/SCID小鼠移植试验中表现致瘤性；在使用了Notch通路阻断剂（γ-分泌酶抑制剂，GSI）后，Notch通路下游信号表达减弱，移植瘤无法在NOD/SCID小鼠中再植，形成新的移植瘤；其研究结果表明，高Notch活性与致瘤性和肿瘤耐药性等有关，其抑制剂有潜在的治疗价值。

### 2.2 Hedgehog通路

Hedgehog通路在胚胎发育中起到重要作用，在成体组织的修复与再生中处于中心地位。Hedgehog通路的调节异常与脑部、皮肤、胃肠道及胰腺的癌症均有联系^[33]^。Hedgehog通路涉及三种配体：Dhh、Shh、Ihh；两类转录因子：Ci、Gli；正常情况下，该通路由Gli1介导的正反馈环和PTCH1，PTCH2，HHIP1介导的负反馈环调控维持稳定，其失调则可能导致癌变^[34]^。Watkins等^[35]^发现Shh和Gli1在肺的气道上皮损伤修复中高度活跃，7种SCLC和7种NSCLC细胞系均表达Shh；对癌症组织的Shh和Gli1检测数据显示，SCLC的组织样本中50%共表达Shh和Gli1，NSCLC则仅10%共表达Shh和Gli1。也有其他研究在40例SCLC样本中检出Gli表达高达85%^[33]^。Katoh等^[34]^发现Hedgehog通路可与下述的Wnt通路共同诱导CD133和CD44等干细胞标志物，促进上皮-间充质转换。

### 2.3 Wnt通路

Wnt通路在多种器官系统的形态发生过程中都扮演了重要角色，参与了多种组织的损伤后再生过程。Wnt通路与组织的癌变也有相关性，在结直肠癌，黑色素瘤等中都存在Wnt异常激活^[36]^。Wnt配体是一类分泌蛋白，其家族中与人类肺癌相关的主要有Wnt1、Wnt2、Wnt7a、Wnt5a^[36]^；Wnt通路可分为β-连环蛋白依赖的经典Wnt通路和β-连环蛋白非依赖的非经典Wnt通路；值得一提的是，根据肺癌类型的不同，非经典通路的异常激活可能是肿瘤抑制因素或肿瘤诱发因素^[37]^，这与Wnt的激活在其他肿瘤的发生中的特点不同。He等^[38]^用阻断Wnt1和Wnt2信号通路的siRNA或单克隆抗体，都成功诱导了NSCLC细胞的凋亡。Yang等^[37]^发现在肺腺癌A549细胞系和顺铂耐药的A549/DDP细胞系中，Wnt5a都能通过其介导的非经典Wnt通路增加ALDH阳性LCSC的干细胞性，实验也证实了Wnt5a/PKC通路与NSCLC的顺铂耐药有关。

### 2.4 其他通路

另有一些研究^[4-7]^显示，除上述几种通路外，EGFR-Notch通路、STAT3通路、JNK通路和PI3K通路等与肺癌的预后不良有关，且伴随一些CSC标志物的表达，提示其在LCSC产生与维持过程中的作用。此外，Oct4，Sox2和Nanog这三种胚胎干细胞自我更新的核心转录因子，都被认为与LCSC自我更新过程有密切关系^[7]^。

## 3 与LCSC相关的治疗策略

### 3.1 针对LCSC标记物的靶向治疗策略

Hu等^[39]^研究发现，麦角黄酮酸D（SAD）可以抑制侧群细胞的肿瘤球形成能力，而其具体机制，则是激活钙蛋白酶1来缩短ABCG2的半衰期，使得ABCG2表达下调，且侧群细胞比例也减少。Niu等^[40]^用低分子量肝素（low molecular weight heparin, LMWH）处理顺铂耐药的A549侧群细胞，发现LMWH对侧群细胞的增殖和凋亡过程无显著影响，但是LMWH极大地降低了侧群细胞的集落形成能力和ABCG2的表达，进一步研究显示，LMWH是通过诱导蛋白酶体介导的ABCG2的降解实现的上述对SP细胞的影响，LMWH与顺铂治疗的结合可有效控制肿瘤的耐药性。To等^[41]^发现，对于高热疗法带来的ABCG2表达上调与耐药性上升，培利替尼（pelitinib）可作为ABCG2的竞争性抑制剂，显著抑制ABCG2介导的药物外排导致的耐药性。Yeh等^[42]^用三氟拉嗪（trifluoperazine）处理LCSC，降低了LCSC的肿瘤球形成能力，下调了CD44和CD133的表达，且三氟拉嗪与吉非替尼（gefitinib）或顺铂结合使用，可有效控制LCSC耐药性。根据ALDH1阳性的LCSC对顺铂治疗有耐药性这一现象，MacDonagh等^[43]^用DEAB和双硫仑两种物质靶向ALDH1，恢复了此类细胞亚群对顺铂药物治疗的敏感性。此外，近年出现了一些利用纳米给药新方法的靶向治疗思路，如Huang等^[44]^将吉非替尼（gefitinib）装载到带有CD133特异性寡核苷酸适配子的纳米胶束上，可将药物靶向输送到CD133阳性LCSC部位，体外实验中显著降低了CD133阳性细胞在肿瘤细胞中的比例，且药物抑制肿瘤球形成。

### 3.2 针对LCSC信号通路的靶向治疗策略

Liu等^[45]^从A549细胞系中分离出CD133阳性的LCSC，用γ分泌酶抑制剂DAPT（GSI-Ⅸ）阻断Notch通路，与顺铂治疗相结合，发现GSI显著增强了顺铂的疗效；Ma等^[46]^在H520细胞系中用GSI阻断Notch3观察到类似的效果，且Ma等用特异的siRNA抑制了Notch3通路后，A549和H520的克隆产生能力和肿瘤球形成能力都明显降低。

Katoh等^[34]^的研究指出，针对Hedgehog通路的癌症治疗策略，Hedgehog通路抑制剂应与RTK通路抑制剂和GPCR通路调节剂联合使用，因为后两者与Hedgehog的激活与抑制密切相关。Zhu等^[47]^发现姜黄素（Curcumin）可同时抑制Wnt通路及Shh介导的Hedgehog通路的激活，在体外实验中抑制肿瘤球形成，降低CSC的标志物的表达，诱导CSC的凋亡。Ahmad等^[48]^证实Hedgehog通路与NSCLC的上皮-间充质转化（EMT）过程和埃罗替尼、顺铂耐药性有关；研究还表明，针对Hedgehog通路的特异siRNA和该通路的拮抗剂GDC-0449都诱导了CSC标志物的下调，并使得EMT细胞恢复对药物的敏感性；另一项研究^[49]^中将Hedgehog拮抗剂SANT-1与吉非替尼（gefitinib）共同使用，也明显抑制了对EGFR-TKI耐药的NSCLC细胞致瘤性。

Yang等^[37]^对Wnt5a/Ca^2+^/PKC通路加入PKC抑制剂GF109203X后，观察到顺铂耐药的肺癌A549/DDP细胞系凋亡数目增加，显示了其对化疗抵抗的NSCLC患者的治疗价值。Zhang等^[50]^从SPC-A1和PC9细胞系中分离得到LCSC后，用Pyrvinium Pamoate（PP）与其他三种Wnt通路的抑制剂（沙利霉素，ICG-001和水飞蓟宾）作为对照进行肿瘤细胞存活试验，发现PP可选择性地抑制LCSC集落形成，下调多潜能干细胞信号通路的表达水平，其半数最大抑制浓度远低于后三者。You等^[51]^制备了Wnt-2蛋白N末端的单克隆抗体（mAb），并证实其可诱导高表达Wnt-2的NSCLC细胞系凋亡，对缺乏Wnt-2表达的正常气道组织细胞则不诱导凋亡；同时，靶向*Wnt*-2基因转录的mRNA的siRNA也成功诱导了NSCLC细胞系的凋亡；研究还发现，在mAb和siRNA诱导的NSCLC中均观察到了生存素蛋白表达下调，提示该蛋白在Wnt通路中的重要性，或可作为治疗靶点。

### 3.3 抑制上皮-间充质转化

EMT对于癌细胞的迁徙与入侵有着重要的作用，LCSC的一些异常信号通路会促进肺癌的EMT过程激活^[31, 34]^，因此抑制EMT通路的激活也是一种潜在的治疗策略。Met通路对EMT有促进作用，PHA-665752和PF-2341066（克唑替尼，crizotinib）可阻断Met的磷酸化过程，逆转SCLC的耐药性；对H69M细胞系，体外实验分别用依托泊苷（Etoposide），克唑替尼以及两者联合进行治疗，结果表明两者联合对Met的抑制作用更强，小鼠体内实验亦证实两者联合对肿瘤生长的抑制效果比各自单独使用更为明显，提示克唑替尼恢复了已产生耐药性的癌细胞对药物的敏感性^[52, 53]^。Li等^[54]^设计并合成了一种具有双重特异性的抗体（BsAb-5），靶向CD166阳性的LCSC中的c-MET（细胞-间质上皮转化因子）及CTLA-4（细胞毒T细胞相关蛋白4）双重位点，体外实验显示c-MET-Notch通路被抑制，且BsAb-5使小鼠的移植瘤体积明显缩小。

### 3.4 针对LCSC的免疫疗法

上述各种针对LCSC的靶向治疗策略均涉及到小分子抑制剂、单克隆抗体和RNA等，事实上近年来针对CSC的疫苗疗法也有一定进展。Lu等^[55]^利用黑素瘤和鳞状细胞癌的CSC裂解产物冲击致敏树突状细胞（DC），这种CSC-DC疫苗能限制小鼠的肿瘤生长，减少肺转移，延长生存期，并证实该冲击过程与CSC-DC启动的B细胞分泌IgG，靶向高表达ALDH的CSC的有关；该疫苗显著减少了原发瘤中的CSC数目，对于传统抗癌治疗后的局部或全身性复发可能有重要的临床价值。此外，目前针对NSCLC和SCLC的各种疫苗大都基于肿瘤相关抗原引起的免疫应答，这些经验将会极大地推动LCSC相关免疫疗法的发展^[56, 57]^。

## 4 结论与展望

肺癌干细胞的研究已经积累了较多的资料，但LCSC的一些生物学特性尚未透彻：不同类型肺癌的CSC的分辨特征的差异，逃避放化疗的具体机制以及其自我更新功能的推动因素等。并且，NSCLC的异质性导致其CSC缺乏具有普遍鉴别作用的特异性标志物，这将对LCSC的概念运用于肺癌治疗造成巨大的障碍。虽然上述一些药物在体外实验和动物实验中显示出非常有效的杀灭或抑制LCSC的作用，但仍需大量的工作去评估其对人体正常细胞的毒害作用，并且需要设法尽量降低其毒副作用才有望在临床上使用。毋庸置疑地是，肿瘤治疗需要多种治疗策略相互配合，对肺癌来说，针对LCSC的策略只是其中一种前景广阔的思路。

## References

[1] Siegel RL, Miller KD, Jemal A (2016). Cancer statistics, 2016. CA Cancer J Clin.

[b2] 2Wicha MS, Liu S, Dontu G. Cancer stem cells: an old idea--a paradigm shift. Cancer Res, 2006, 66(4): 1883-1890. discussion 1895-1896. doi: 10.1158/0008-5472.CAN-05-3153

[3] Reya T, Morrison SJ, Clarke MF (2001). Stem cells, cancer, and cancer stem cells. Nature.

[4] Sullivan James P, Minna John D, Shay Jerry W (2010). Evidence for self-renewing lung cancer stem cells and their implications in tumor initiation, progression, and targeted therapy. Cancer and Metastasis Reviews.

[5] Leeman K T, Fillmore C M, Kim CF (2014). Lung stem and progenitor cells in tissue homeostasis and disease. Curr Top Dev Biol.

[6] Amber L, Barbara D (2013). Lung cancer stem cells: Progress, prospects. Cancer Letters.

[7] Singh S, Chellappan S (2014). Lung cancer stem cells: Molecular features, therapeutic targets. Mol Aspects Med.

[8] Wu X, Chen H, Wang X (2012). Can lung cancer stem cells be targeted for therapies?. Cancer Treatment Rev.

[9] Frank NY, Schatton T, Frank MH (2010). The therapeutic promise of the cancer stem cell concept. J Clin Invest.

[10] Chen YC, Hsu HS, Chen YW (2008). Oct-4 expression maintained cancer stem-like properties in lung cancer-derived CD133-positive cells. PLoS One.

[11] Miraglia S, Godfrey W, Yin AH (1997). A novel five-transmembrane hematopoietic stem cell antigen: isolation, characterization, molecular cloning. Blood.

[12] Tirino V, Camerlingo R, Franco R (2009). The role of CD133 in the identification, characterisation of tumour-initiating cells in non-small-cell lung cancer. Eur J Cardiothorac Surg.

[13] Hilbe W, Dirnhofer S, Oberwasserlechner F (2004). CD133 positive endothelial progenitor cells contribute to the tumour vasculature in non-small cell lung cancer. J Clin Pathol.

[14] Eramo A, Lotti F, Sette G (2008). Identification and expansion of the tumorigenic lung cancer stem cell population. Cell Death Differ.

[15] Yoshida A, Hsu L C, Dave V (1992). Retinal oxidation activity and biological role of human cytosolic aldehyde dehydrogenase. Enzyme.

[16] Ginestier C, Hur M H, Charafe-Jauffret E (2007). ALDH1 is a marker of normal and malignant human mammary stem cells and a predictor of poor clinical outcome. Cell Stem Cell.

[17] Moreb J S, Zucali J R, Ostmark B (2007). Heterogeneity of aldehyde dehydrogenase expression in lung cancer cell lines is revealed by Aldefluor flow cytometry-based assay. Cytometry B Clin Cytom.

[18] Patel M, Lu L, Zander D S (2008). ALDH1A1 and ALDH3A1 expression in lung cancers: correlation with histologic type and potential precursors. Lung Cancer.

[19] Ucar D, Cogle C R, Zucali J R (2009). Aldehyde dehydrogenase activity as a functional marker for lung cancer. Chem Biol Interact.

[20] Jiang F, Qiu Q, Khanna A (2009). Aldehyde dehydrogenase 1 is a tumor stem cell-associated marker in lung cancer. Mol Cancer Res.

[21] Hu J, Li J, Yue X (2017). Expression of the cancer stem cell markers ABCG2 and OCT-4 in right-sided colon cancer predicts recurrence and poor outcomes. Oncotarget.

[22] Zhang G, Wang Z, Luo W (2013). Expression of potential cancer stem cell marker ABCG2 is associated with malignant behaviors of hepatocellular carcinoma. Gastroenterol Res Pract.

[23] Jiang Y, He Y, Li H (2012). Expressions of putative cancer stem cell markers ABCB1, ABCG2, and CD133 are correlated with the degree of differentiation of gastric cancer. Gastric Cancer.

[24] Ho MM, Ng AV, Lam S (2007). Side population in human lung cancer cell lines, tumors is enriched with stem-like cancer cells. Cancer Res.

[25] Sung JM, Cho HJ, Yi H (2008). Characterization of a stem cell population in lung cancer A549 cells. Biochem Biophys Res Commun.

[26] Liu Z, Zhang J, Kang H (2016). Significance of stem cell marker Nanog gene in the diagnosis, prognosis of lung cancer. Oncol Lett.

[27] Silva J, Nichols J, Theunissen TW (2009). Nanog is the gateway to the pluripotent ground state. Cell.

[28] Leung EL, Fiscus RR, Tung JW (2010). Non-small cell lung cancer cells expressing CD44 are enriched for stem cell-like properties. PLoS One.

[29] Zhang WC, Shyh-Chang N, Yang H (2012). Glycine decarboxylase activity drives non-small cell lung cancer tumor-initiating cells, tumorigenesis. Cell.

[30] Westhoff B, Colaluca I N, D' ario G (2009). Alterations of the Notch pathway in lung cancer. Proc Natl Acad Sci U S A.

[31] Yuan X, Wu H, Han N (2014). Notch signaling and EMT in non-small cell lung cancer: biological significance and therapeutic application. J Hematol Oncol.

[32] Hassan KA, Wang L, Korkaya H (2013). Notch pathway activity identifies cells with cancer stem cell-like properties, correlates with worse survival in lung adenocarcinoma. Clin Cancer Res.

[33] Velcheti V, Govindan R (2007). Hedgehog signaling pathway and lung cancer. J Thorac Oncol.

[34] Katoh Y, Katoh M (2009). Hedgehog target genes: mechanisms of carcinogenesis induced by aberrant hedgehog signaling activation. Curr Mol Med.

[35] Watkins DN, Berman DM, Burkholder SG (2003). Hedgehog signalling within airway epithelial progenitors and in small-cell lung cancer. Nature.

[36] He B, Barg RN, You L (2005). Wnt signaling in stem cells, non-small-cell lung cancer. Clin Lung Cancer.

[37] Yang J, Zhang K, Wu J (2016). Wnt5a increases properties of lung cancer stem cells, resistance to cisplatin through activation of Wnt5a/PKC signaling pathway. Stem Cells Int.

[38] He B, You L, Uematsu K (2004). A monoclonal antibody against Wnt-1 induces apoptosis in human cancer cells. Neoplasia.

[39] Hu Y P, Tao L Y, Wang F (2013). Secalonic acid D reduced the percentage of side populations by down-regulating the expression of ABCG2. Biochem Pharmacol.

[40] Niu Q, Wang W, Li Y (2012). Low molecular weight heparin ablates lung cancer cisplatin-resistance by inducing proteasome-mediated ABCG2 protein degradation. PLoS One.

[41] To KK, Poon DC, Wei Y (2015). Pelitinib (EKB-569) targets the up-regulation of ABCB1, ABCG2 induced by hyperthermia to eradicate lung cancer. Br J Pharmacol.

[42] Yeh C T, Wu A T, Chang P M (2012). Trifluoperazine, an antipsychotic agent, inhibits cancer stem cell growth, overcomes drug resistance of lung cancer. Am J Respir Crit Care Med.

[43] Macdonagh L, Gallagher MF, Ffrench B (2017). Targeting the cancer stem cell marker, aldehyde dehydrogenase 1, to circumvent cisplatin resistance in NSCLC. Oncotarget.

[44] Huang X, Huang J, Leng D (2017). Gefitinib-loaded DSPE-PEG2000 nanomicelles with CD133 aptamers target lung cancer stem cells. World J Surg Oncol.

[45] Liu J, Mao Z, Huang J (2014). Blocking the NOTCH pathway can inhibit the growth of CD133-positive A549 cells, sensitize to chemotherapy. Biochem Biophys Res Commun.

[46] Ma Y, Li M, Si J (2016). Blockade of Notch3 inhibits the stem-like property and is associated with ALDH1A1 and CD44 via autophagy in non-small lung cancer. Int J Oncol.

[47] Zhu JY, Yang X, Chen Y (2017). Curcumin Suppresses Lung Cancer Stem Cells via Inhibiting Wnt/beta-catenin and Sonic Hedgehog Pathways. Phytother Res.

[48] Ahmad A, Maitah MY, Ginnebaugh KR (2013). Inhibition of Hedgehog signaling sensitizes NSCLC cells to standard therapies through modulation of EMT-regulating miRNAs. J Hematol Oncol.

[49] Bai XY, Zhang XC, Yang SQ (2016). Blockade of hedgehog signaling synergistically increases sensitivity to epidermal growth factor Receptor tyrosine kinase inhibitors in non-small-cell lung cancer cell lines. PLoS One.

[50] Zhang X, Lou Y, Zheng X (2015). Wnt blockers inhibit the proliferation of lung cancer stem cells. Drug Des Devel Ther.

[51] You L, He B, Xu Z (2004). Inhibition of Wnt-2-mediated signaling induces programmed cell death in non-small-cell lung cancer cells. Oncogene.

[52] Canadas I, Rojo F, Taus A (2014). Targeting epithelial-to-mesenchymal transition with Met inhibitors reverts chemoresistance in small cell lung cancer. Clin Cancer Res.

[53] Ou SH, Bazhenova L, Camidge DR (2010). Rapid and dramatic radiographic and clinical response to an ALK inhibitor (crizotinib, PF02341066) in an ALK translocation-positive patient with non-small cell lung cancer. J Thorac Oncol.

[54] Li JF, Niu YY, Xing YL (2017). A novel bispecific c-MET/CTLA-4 antibody targeting lung cancer stem cell-like cells with therapeutic potential in human non-small cell lung cancer. Biosci Rep.

[55] Lu L, Tao H, Chang AE (2015). Cancer stem cell vaccine inhibits metastases of primary tumors, induces humoral immune responses against cancer stem cells. Oncoimmunology.

[56] Freeman-Keller M, Goldman J, Gray J (2015). Vaccine immunotherapy in lung cancer: Clinical experience, future directions. Pharmacol Ther.

[57] Cuppens K, Vansteenkiste J (2014). Vaccination therapy for non-small-cell lung cancer. Curr Opin Oncol.

